# Cerebrospinal fluid profiles of targeted metabolomics on neurotransmitters in patients with post-neurosurgical bacterial meningitis

**DOI:** 10.3389/fcimb.2025.1484144

**Published:** 2025-02-25

**Authors:** Zhaojun Mei, Liao Guan, Ziao Xu, Hongwei Cheng, Lei Ye

**Affiliations:** ^1^ Department of Neurosurgery, The First Affiliated Hospital of Anhui Medical University, Hefei, China; ^2^ Department of Neurosurgery, Zhenjiang First People’s Hospital, Jiangsu, Zhenjiang, China

**Keywords:** neurotransmitter, bacterial meningitis, neurosurgery, hemorrhagic stroke, tyrosine metabolism

## Abstract

**Background:**

Post-neurosurgical bacterial meningitis (PNBM) is a severe complication in patients receiving neurosurgical treatments. Pathogens and neuroinflammation have been reported to influence metabolites in the microenvironment of the central nervous system. However, information about the relationship between neurotransmitter levels and PNBM is still limited. In this study, we aimed to investigate the diagnostic potential of neurotransmitters for PNBM in the patients with stroke.

**Methods:**

In this study, a total of 66 stroke patients were recruited. Among them, 40 patients were complicated with PNBM. We profiled cerebrospinal fluid (CSF) levels of neurotransmitter precursors and metabolites using the targeted metabolomics method, which contained 26 precursors and metabolites of neurotransmitters, using ultra-performance liquid chromatography coupled with mass spectrometry (UPLC/MS).

**Results:**

We found that 14 biomarkers were downregulated but 3,4-dihydroxyphenylacetic acid (DOPAC) was upregulated in the CSF of PNBM patients. Among the biomarkers, D-glutamine (AUC=1.000), Boc-D-Tyr-OH (AUC=0.9447), L(+)-arginine (AUC=0.9418), and DOPAC (AUC=0.9173) had strong diagnostic efficiency for PNBM. Bioinformatic analysis showed that tyrosine metabolism, butanoate metabolism, histidine metabolism, alanine, aspartate and glutamate metabolism, glycerophospholipid metabolism, arginine and proline metabolism, and tryptophan metabolism might be involved in the pathogenesis of PNBM. After reviewing previous studies, we found a probable diverse pathophysiological alteration between PNBM and community-acquired bacterial meningitis.

**Conclusions:**

In summary, we identified downregulated levels of D-glutamine, Boc-D-Tyr-OH, L(+)-arginine, and phenprobamate, and an upregulated level of DOPAC in CSF to have strong diagnostic efficiencies. The results also offered potential targets for the treatment of PNBM.

## 1 Introduction

Post-neurosurgical bacterial meningitis (PNBM) is a severe complication in patients receiving neurosurgical treatments, with the reported incidence rate ranging from 0.3% to 10% in different neurosurgical diseases (Blomstedt, 1985; Zhang et al., 2017). Previous studies have demonstrated that stroke-induced immunosuppression increased the risks of systemic and neurological infections (Sarrafzadeh et al., 2011). In an epidemiological study, the incidence rate of PNBM in cerebrovascular disorder was reported to be 4.9% (Zhang et al., 2017). In comparison with community-acquired bacterial meningitis (CABM), the clinical symptoms of PNBM patients are usually concealed by primary diseases or surgery-induced injuries, thus providing limited information to support the diagnosis. Furthermore, the prophylactic application of antibiotics during the perioperative period also has a significant impact on pathogenic identification in cerebrospinal fluid (CSF). So far, an accurate and prompt diagnosis of PNBM is still difficult in clinical practice. Therefore, this study aimed to develop novel biomarkers in CSF with robust diagnostic potential to satisfy the urgent needs in the clinical diagnosis of PNBM.

Current studies have mostly focused on the diagnostic role of neuroinflammation-related cytokines and neurocyte-related biomarkers in central nervous system (CNS) infection, such as TNF-α, IL-1β, and GFAP (Baran et al., 2023; Caragheorgheopol et al., 2023). However, these molecules were also reported to participate in the pathogenesis of stroke. A neurotransmitter is a bioactive chemical that transmits information between neurons or between neurons and effector cells. Previous studies have found that neuroinflammation and infection in the CNS changed the neurophysiological environment and consequently influenced the levels of neurotransmitters (Wilson et al., 2002; Kong et al., 2022). Linthorst et al. (1995) reported that LPS also induced increased levels of extracellular 5-hydroxytryptamine (5-HT) and its metabolite 5-hydroxyindole acetic acid (5-HIAA) in the hippocampus region of rats. Furthermore, because neurotransmitter changes in the CNS not only reflect the pathological condition but also indicate potential neurological deficit, it might be theoretically considered a candidate biomarker in neurological disorders. However, we only found one clinical study concerning the association between community-acquired meningitis and CSF neurotransmitters.

However, in PNBM, the pathological process of infection might be diverse because of its secondary insult characteristics to the brain compared to CABM. Therefore, the neurotransmitter profile has diagnostic potential for PNBM. Moreover, it may also help the understanding of pathophysiological alterations of neural circuits in the complicated coincidence of primary neurological disease with secondary infection.

In this study, we performed a targeted metabolomics analysis of neurotransmitter precursors and metabolites in CSF samples from patients with stroke and aimed to analyze the diagnostic potential of neurotransmitters in PNBM. Furthermore, this study also offers information on the potential mechanisms of neural circuit changes in the pathological condition of PNBM.

## 2 Materials and methods

### 2.1 Participants and sample collection

A total of 66 patients with hemorrhagic stroke were recruited in this study. All patients were from the Chinese Han population. The categories of disease included intracerebral hemorrhage and subarachnoid hemorrhagic and cerebrovascular malformations. The diagnosis was confirmed by two senior neurosurgeons with supporting evidence from neuro-imaging tests. Lumbar cistern drainage operations were performed for all patients to obtain CSF. All patients underwent laboratory tests of CSF characteristics for potential infectious signs, such as hyperpyrexia, signs of meningeal irritation, or altered consciousness and mental states. Among all the participants, 40 patients were categorized as PNBM based on the diagnostic criteria issued by the Infectious Disease Society of America’s (IDSA’s) Clinical Practice Guidelines for Healthcare-Associated Ventriculitis and Meningitis 2017 and the Chinese Expert Consensus of Diagnostic and Therapy for the Neurosurgical Central Nervous System Infections in 2021. According to the guidelines, PNBM diagnosis relies on either positive results from Gram’s staining/bacterial culture or the CSF indications (simultaneously satisfying CSF white blood cells > 100x10^6^/L, CSF glucose< 2.2 mmol/L, and CSF-to-blood glucose ratio < 0.4). Otherwise, if none or 1 of 3 indicators reached abnormal levels, and the infectious symptoms were resolved without receiving antibiotics, we considered the patient to be PNBM-free. Among these patients, six patients had positive results for bacterial cultures, three of whom were infected with *Stenotrophomonas maltophilia*, *Moderate thermophiles*, and *Streptococcus agalactiae*, respectively. Two patients were infected with *Acinetobacter baumannii*, and one patient was jointly infected with *Pseudomonas aeruginosa* and *Aeromonas caviae*. Finally, 26 patients were included in the PNBM-free group. The inclusion criteria were: 1) patients with neurosurgical treatments; 2) patients with complete demographic and clinicopathological data; and 3) adequate amount and quality of CSF samples available for metabolomics analysis. The exclusion criteria were: 1) patients with concomitant or complicated with other types of CNS disorders (e.g., brain tumors, neurodegenerative disorders, seizure, or psychiatric disorders); 2) unqualified or inadequate amount of CSF samples available for the study (e.g., severely hemolyzed or contaminated sample during transportation or storage; 3) patients who had systemic inflammatory diseases or malignant tumors. CSF samples were extracted via lumbar cistern drainage or lumbar puncture using the aseptic technique when the patients were suspected to have a CNS infection. For the targeted metabolomics test, a volume of 50 μl was needed for each sample. After the sample collection, all CSF samples were centrifuged at 3,000 rpm for 15 min. Before the supernatant was frozen at -80°C, all samples were tested for the total protein concentration. In order to avoid protein degradation, we also tested the total protein concentration prior to the omic-related analysis if the storage time was longer than 3 months. The demographic data of the patients and biochemical characteristics of the CSF samples are summarized in **Table 1**.

**Table 1:** Clinicopathological characteristics of the patients recruited in this study.

		PNBM (n=40)	Non-PNBM (n=26)	P value
Age (years, mean ± SD)		50.03 ± 15.46	53.58 ± 15.94	0.371
Sex				0.760
	Male	20	12	
	Female	20	14	
CSF				
	Glucose (mmol/L, mean ± SD)	2.01 ± 0.93	4.42 ± 0.42	**<0.001**
	Protein (g/L, IQR)	2.12 [1.10, 3.50]	0.63 [0.40, 5.75]	**<0.001**
	White blood cells (x10^6^/L, IQR)	1054 [224, 2372]	34 [6, 293]	**<0.001**
	Chlorine (mmol/L, mean ± SD)	119.9 ± 7.9	127.2 ± 6.4	**<0.001**
Blood glucose (mmol/L, mean ± SD)		6.59 ± 3.33	6.89 ± 2.43	0.689
cGlu/bGlu ratio (mean ± SD)		0.34 ± 0.17	0.69 ± 0.17	**<0.001**

PNBM, post-neurosurgical bacterial meningitis; CSF, cerebrospinal fluid; cGlu/bGlu ratio, CSF to blood glucose ratio; IQR, interquartile range.

The p value in bold means the statistical significance.

### 2.2 Metabolomics analysis

The metabolomics analysis was formed in Sinotech Genomics Biotechnology Co. Ltd. and referenced with an established protocol (Cheng et al., 2021). Briefly, after the transient centrifugation, the supernatants (50 μL) were used to measure the neurotransmitter contents using the Ultra Performance Liquid Chromatography-Triple Quadrupole Mass Spectrometry (UPLC-QQQ-MS) method (AB SCIEX 5500 QQQ Mass Spectrometry) in multiple reaction monitoring (MRM) mode. Sample analysis was carried out with an Acquity UPLC system (Waters Corp., Milford, MA, USA). The LC separations were carried out on Acquity UPLC HSS T3 columns (100 × 2.1 mm, 1.8 μm) with a flow rate of 0.2 mL/min, an injection volume of 5 µL, and the column temperature was 40°C. Mobile phase A consisted of 0.1% aqueous formic acid in water. Mobile phase B was acetonitrile. Mass spectrometry was performed as follows: curtain gas, 35 arb (arbitrary units); collision gas, 9 arb; ion source gas, 55 arb; ion spray voltage, 4500 V; ion source temperature, 550°C. We used MultiQuant software to extract and preprocess the data. The standard solution was added to the sample vial to quantify and identify peaks of metabolites.

The metabolite package of neurotransmitters included L-glutamic acid, D-glutamine, L-histidine, Boc-D-Tyr-OH, L(+)-arginine, D-tryptophan, 5-HIAA, γ-aminobutyric acid, histamine dihydrochloride, DL-adrenalin, norepinephrine, serotonin, dopamine, 5-hydroxytryptophan, D-kynurenine, kynurenic acid, tyramine, tryptamine, 3-methoxytyramine (3-MT), acetyl choline, choline, DL-metanephrine, melatonin, vanilmandelic acid, homovanillic acid (HVA), and 3,4-dihydroxyphenylacetic acid (DOPAC). However, due to a technique fault, we failed to detect four of these molecules, including histamine dihydrochloride, DL-adrenalin, melatonin, and vanilmandelic acid. Therefore, 22 molecules were finally included in the analysis.

### 2.3 Statistical analysis

All statistical analyses were performed using SPSS software (19.0 version, IBM, USA). All continuous data were depicted as mean ± standard deviation if the data fitted a normal distribution (mean ± SD) and then analyzed with Student’s *t*-test. Otherwise, it was presented as medians with interquartile range (IQR) and then analyzed using the Mann–Whitney U test. In consideration of the limited sample size, we employed Bayesian discriminant analysis to process the data, leveraging prior information to enhance robustness and accuracy. Binary data were analyzed using the chi-squared test. In the correlation analysis among the molecules in CSF, Pearson’s correlation coefficients were assessed for each molecule. Receiver operating characteristic (ROC) curve analysis was conducted to assess the diagnostic efficacies of molecules in PNBM via the index of area under the curve (AUC). Bootstrap methods were used in the ROC analysis. P<0.05 was considered to indicate statistical significance.

## 3 Results

### 3.1 Cohort description and quality control

We performed a quality control evaluation of the metabolomics analysis with principal component analysis (PCA). PCA is a statistical method that converts a set of observed possible correlated variables into linear uncorrelated variables (i.e., principal components) using orthogonal transformation. PCA can reveal the internal structure of the data to better explain the data variables. Metabolome data can be considered a multivariate data set that can be displayed in a high-dimensional data space coordinate system. PCA can provide a relatively low-dimensional image of this data set (two-dimensional or three-dimensional) and the display is the “projection” of the original object at the point containing the most information, effectively using a small number of principal components to reduce the dimension of the data. The scores of the horizontal and vertical coordinates of PC1 and PC2, which represented the scores of the first and second principal components, were 0.354 and 0.180, respectively (**Supplementary Figure 1A**). Furthermore, metabolomics data have the characteristics of high dimension (a large number of metabolites detected) and small sample size (a small number of detection samples). Among these variables, there are not only differential variables related to categorical variables but also a large number of non-differential variables that may be correlated with each other. This means that if we use a PCA model for analysis, the differential variables will be spread out over more principal components due to the influence of the relevant variables, making it impossible to perform better visualization and subsequent analysis. Therefore, we adopted the OPLS-DA statistical method to analyze the results. Through OPLS-DA analysis, we could filter out the orthogonal variables in metabolites that were not related to categorical variables, and analyze the non-orthogonal variables and orthogonal variables respectively, so as to obtain more reliable information about the difference between groups of metabolites and the degree of correlation of the experimental group. The results showed that the component could be divided into different groups, indicating that the metabolomic results were reliable (**Supplementary Figure 1B**).

### 3.2 CSF metabolomic changes in PNBM patients

**Figure 1:**
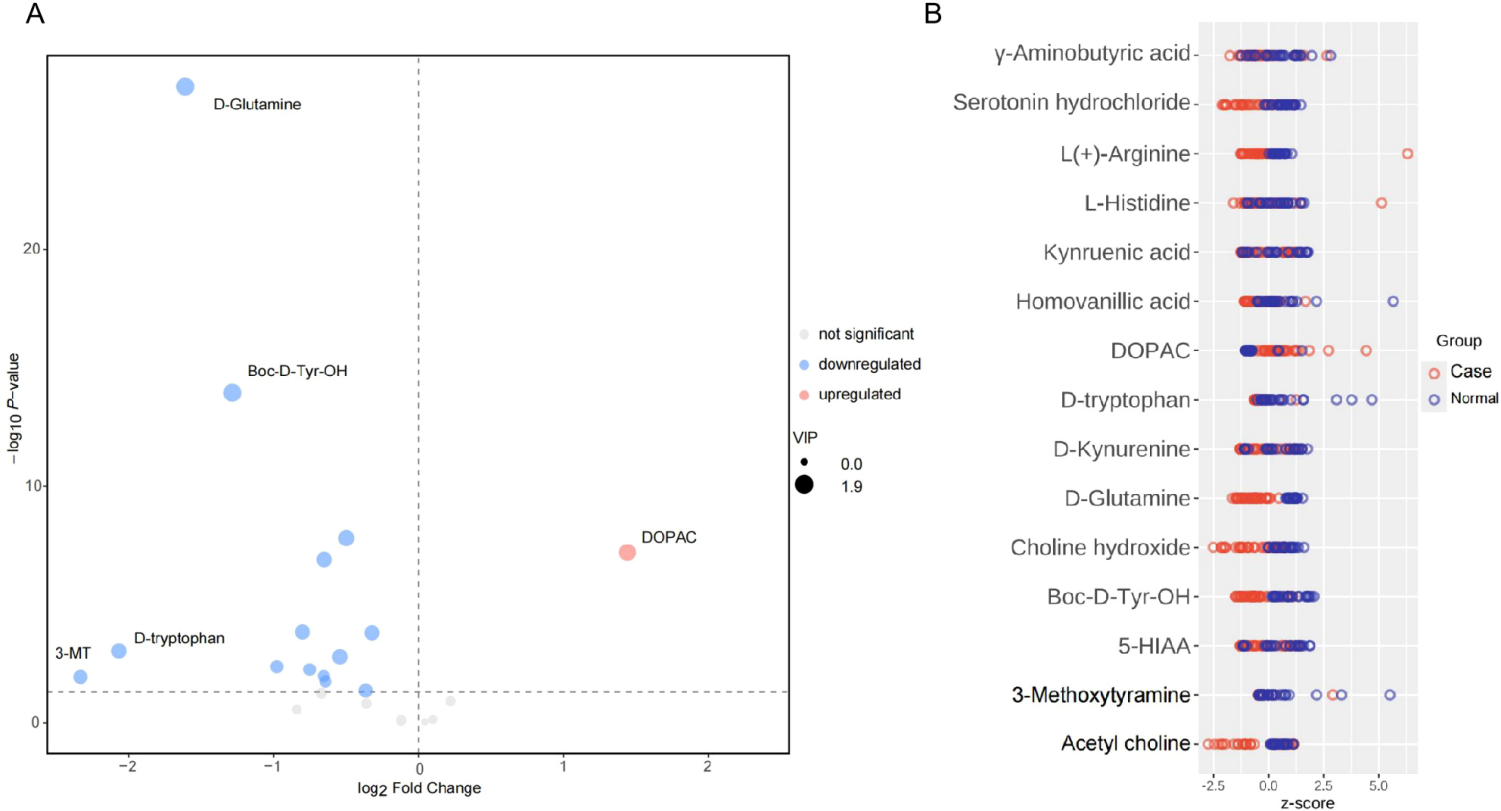
Expression levels of neurotransmitters in CSF between PNBM cases and infection-free subjects. **(A)** In the volcano plot, the red plot indicates the upregulation of CSF neurotransmitters in the PNBM group, while the blue plots indicate the downregulation in the PNBM. **(B)** The Z score plot shows the measurement of the relative content of metabolites at the same level.

In consideration of confounders, there were no differences in age and sex between the groups. Therefore, we thought the distributions of age and sex were balanced between the two groups and it was not necessary to adjust for age and gender when calculating the difference between the two sets of continuous variables. Therefore, we analyzed the neurotransmitter levels in the non-PNBM group between different sexes (male vs. female) and different ages (aged group >60 yo vs. young group ≤ 60 yo). We also found there were no differences for all the neurotransmitters between these groups (**Supplementary Figure 2**). We compared the distribution of different metabolites between the two groups using a volcano plot. Among these, there were 14 downregulated metabolites and just one upregulated metabolite in the CSF samples. The downregulated metabolites included D-glutamine [p = 1.362x10^-27^, log(FC) = -1.609], L-histidine [p = 0.042, log(FC) = -0.363], Boc-D-Tyr-OH [p = 1.149x10^-14^, log(FC) = -1.284], L(+)-arginine [p = 1.419x10^-4^, log(FC) = -0.800], D-tryptophan [p = 9.162x10^-4^, log(FC) = -2.066], 5-HIAA [p = 0.010, log(FC) = -0.652], γ-aminobutyric acid [p = 0.001, log(FC) = -0.542], serotonin [p = 1.248x10^-7^, log(FC) = -0.650], D-kynurenine [p = 0.017, log(FC) = -0.641], kynurenic acid [p = 0.006, log(FC) = -0.749], 3-MT [p = 0.011, log(FC) = -2.330], acetyl choline [p = 1.550x10^-4^, log(FC) = -0.320], choline [p = 1.560x10^-8^, log(FC) = -0.496], and homovanillic acid [p = 0.004, log(FC) = -0.976]. However, the upregulated metabolite was DOPAC [p = 6.264x10^-8^, log(FC) = 1.443] (**Figure 1A**). There were four biomarkers for which the |log2(FC)| was greater than 1.4: D-glutamine, D-tryptophan, 3-MT, and DOPAC. After adjusting for age and sex, the results remained significant. We also used a Z-score plot to illustrate the relative content of metabolites at the same level (**Figure 1B**). Given that age and sex might influence neurotransmitter levels, the statistical results were adjusted by age and sex, and the results remained significant (p < 0.001 for D-glutamine; p = 0.045 for L-histidine; p < 0.001 Boc-D-Tyr-OH; p = 0.001 for L(+)-arginine; p <0.001 for D-tryptophan; p = 0.006 for 5-HIAA; p = 0.001 γ-aminobutyric acid; p <0.001 for serotonin; p = 0.010 for D-kynurenine; p = 0.002 for kynurenic acid; p = 0.002 for 3-MT; p = 0.001 for acetyl choline; p < 0.001 for choline; p = 0.001 for homovanillic acid; and p < 0.001 for DOPAC).

### 3.3 Spearman’s correlation analysis of the metabolites in the CSF samples

**Figure 2:**
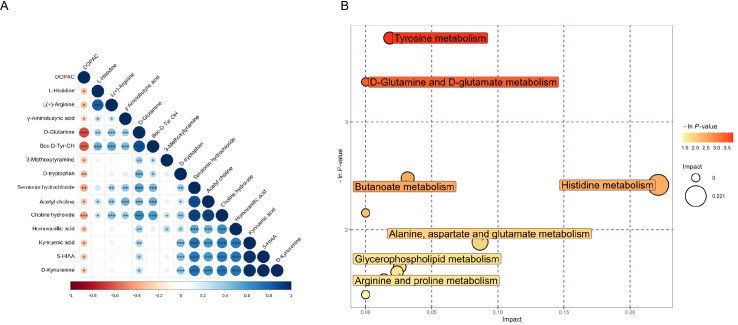
Bioinformatic analysis of neurotransmitters for PNBM. **(A)** Correlation analysis shows the relationships among the neurotransmitters. **(B)** KEGG pathway analysis indicates the potential pathways involved in the pathogenesis of PNBM.

We performed Pearson’s correlation analysis to observe correlations among the metabolites. Although there were abundant correlations among CSF metabolites, we just listed the metabolite pairs with moderate-to-strong correlations (r<-0.08 or r>0.08). We found that D-glutamine was positively correlated with Boc-D-Tyr-OH (r = 0.853, *p* = 1.021x10^-19^). L-histidine was positively correlated with L(+)-arginine (r = 0.818, *p* = 5.383x10^-17^). 5-HIAA was positively correlated with D-kynurenine (r = 0.994, *p* = 2.937x10^-62^) and kynurenic acid (r = 0.969, *p* = 1.267x10^-40^). Serotonin was positively correlated with acetyl choline (r = 0.874, *p* = 1.030x10^-21^) and choline (r = 0.907, *p* = 9.253x10^-26^). D-kynurenine was positively correlated with kynurenic acid (r = 0.971, *p* = 1.059x10^-41^). Acetyl choline was positively correlated with choline (r = 0.918, *p* = 2.170x10^-27^) (**Figure 2A**).

### 3.4 Potential KEGG pathway analysis for PNBM

According to the positive results of the CSF metabolites that had significant associations with PNBM, we performed a bioinformatic analysis to investigate potential pathways involved in the pathogenesis of PNBM. The results showed the potential involvement of seven pathways in the pathogenesis of PNBM, including tyrosine metabolism [-ln(p) = 3.778, impact = 0.018], butanoate metabolism [-ln(p) = 2.477, impact = 0.032]; histidine metabolism [-ln(p) = 2.415, impact = 0.221]; alanine, aspartate and glutamate metabolism [-ln(p) = 1.886, impact = 0.087]; glycerophospholipid metabolism [-ln(p) = 1.656, impact = 0.026]; arginine and proline metabolism [-ln(p) = 1.538, impact = 0.024]; and tryptophan metabolism [-ln(p) = 1.538, impact = 0.014] (**Figure 2B**).

### 3.5 Diagnostic efficiency of neurotransmitter-related biomarkers in PNBM

**Figure 3:**
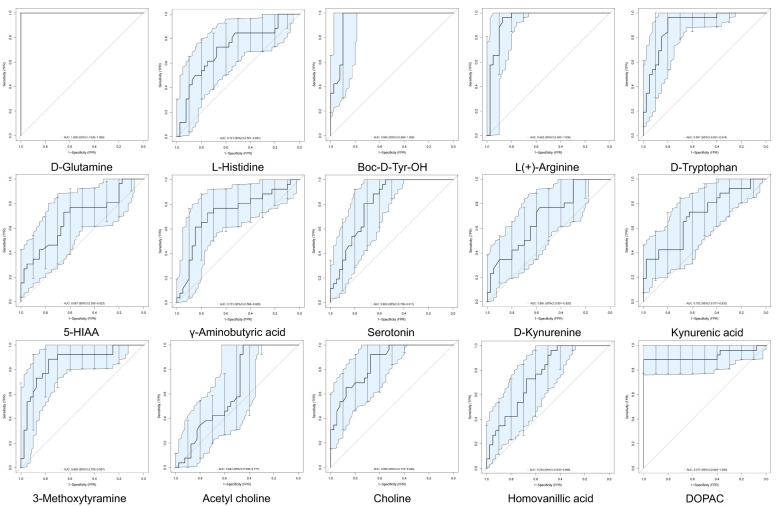
Clinical diagnostic efficiency of neurotransmitters in PNBM with ROC analysis. The area under the curve (AUC) > 0.9 shows a strong diagnostic value for PNBM.

We performed ROC analysis to determine the diagnostic efficiencies of neurotransmitter-related biomarkers in PNBM. The results indicated that D-glutamine (AUC=1.000), Boc-D-Tyr-OH (AUC=0.9447), L(+)-arginine (AUC=0.9418), and DOPAC (AUC=0.9173) had strong diagnostic efficiency for PNBM among the stroke patients (**Figure 3**).

## 4 Discussion

In this study, we performed a targeted metabolomics analysis of neurotransmitter precursors and metabolites in CSF. The results indicated that 14 CSF neurotransmitters were downregulated but DOPAC was upregulated in patients with PNBM. These neurotransmitters were mainly involved in the pathways of tyrosine metabolism; butanoate metabolism; histidine metabolism; alanine, aspartate, and glutamate metabolism; glycerophospholipid metabolism; arginine and proline metabolism; and tryptophan metabolism.

Previous studies have demonstrated that many neurotransmitters are produced by the commensal bacteria in the gastrointestinal tract, such as dopamine, 5-HT, and GABA. We thought pathogenic bacteria in the CNS might not directly produce neurotransmitters. Although some evidence has shown that CNS infection probably influences bacterial composition and abundance in the gut, we mainly aimed to investigate the diagnostic potential of neurotransmitters for PNBM. Some biomarker candidates have been presented in clinical and laboratory investigations with diagnostic potential for PNBM, such as lactate acid and cytokines (Zhang et al., 2017; Kul et al., 2020), but there is still a lack of a robust tool for the accurate and timely diagnosis of infection. In our previous studies, we have explored the potential diagnostic roles of glycomics and immunity-related protein biomarkers for PNBM (Ye et al., 2023; Zhao et al., 2023). However, these molecules provided little information about the infection-induced neurological alterations. Neurotransmitters transmit information between neurons and effector cells, inducing nerve impulses and regulating neuronal excitability and inhibition. A pathological condition influences the neurotransmitter levels and neural circuits, consequently leading to alterations in neurological functions (Fiore et al., 2023; Zhang et al., 2023). Meloni et al. (2015) found that poly-arginine and arginine-rich peptides are highly neuroprotective in an animal model of stroke. Furthermore, Rachedi et al. (2024) demonstrated that the catabolism of glutamine and serine sustained proline and glycine anabolism and promoted collagen biosynthesis, consequently controlling pathologic vascular stiffness. Therefore, the alterations in neurotransmitters can not only provide characteristic changes in nerve function of the patients with PNBM but also serve as a treatment basis.

Irazuzta et al. (1999) reported that bacterial meningitis that was induced by *Group B Streptococcus type III* in rabbits upregulated the levels of glutamate and aspartate in CSF samples. These excitatory neurotransmitters increased neuronal stress and led to excitotoxic effects on the brain. Furthermore, Taj and Jamil (2017) found infection-induced significant changes in the catecholamine level (including DA, DOPAC, and HVA) in the brains of rats. Moreover, they validated the results in clinical samples (Taj and Jamil, 2018) and found the absolute levels of DA, DOPA, HVA, and 5-HIAA in CSF were elevated in the patients with CNS infections with *Neisseria meningitidis*, *Listeria monocytogenes*, and herpes simplex virus, respectively. Furthermore, the upregulations of glutamine and arginine in the CSF were also reported in patients with tuberculous meningitis (Nayak and Bhat, 2005; Parihar et al., 2022). In our study, we found that the levels of 14 neurotransmitter precursors and metabolites in CSF were downregulated but DOPAC was upregulated. The results were discrepant with those of studies in CABM. We hypothesized that the primary neurological disorder also had an impact on the changes in neurotransmitters. In clinical studies, decreased levels of neurotransmitters were reported to be associated with stroke, including dopamine, glutamine, histidine, and L-arginine (Armengou et al., 2003; Lin et al., 2018; Rout et al., 2023). Furthermore, mechanism studies also indicated that these neurotransmitters are involved in the pathogenesis of stroke, even providing a therapeutic effect. Park et al. (2003) used a dopamine infusion method in a neonatal bacterial meningitis animal model and found that dopamine restored the activity of cerebral cortical cell membrane Na^+^ and K^+^-ATPase and decreased lipid peroxidation products. Furthermore, dopamine also helps maintain adequate cerebral perfusion pressure to prevent cerebral ischemia, reduce cerebral energy depletion, and consequently attenuate brain injury. This infers that stroke may also have a significant effect on the metabolism of neurotransmitters and a different pathophysiological characteristic might exist between PNBM and CABM. Therefore, it is necessary to reconsider the applicability of the biomarkers that have been used in clinical routine tests for the diagnosis of CNS infection.

**Figure 4:**
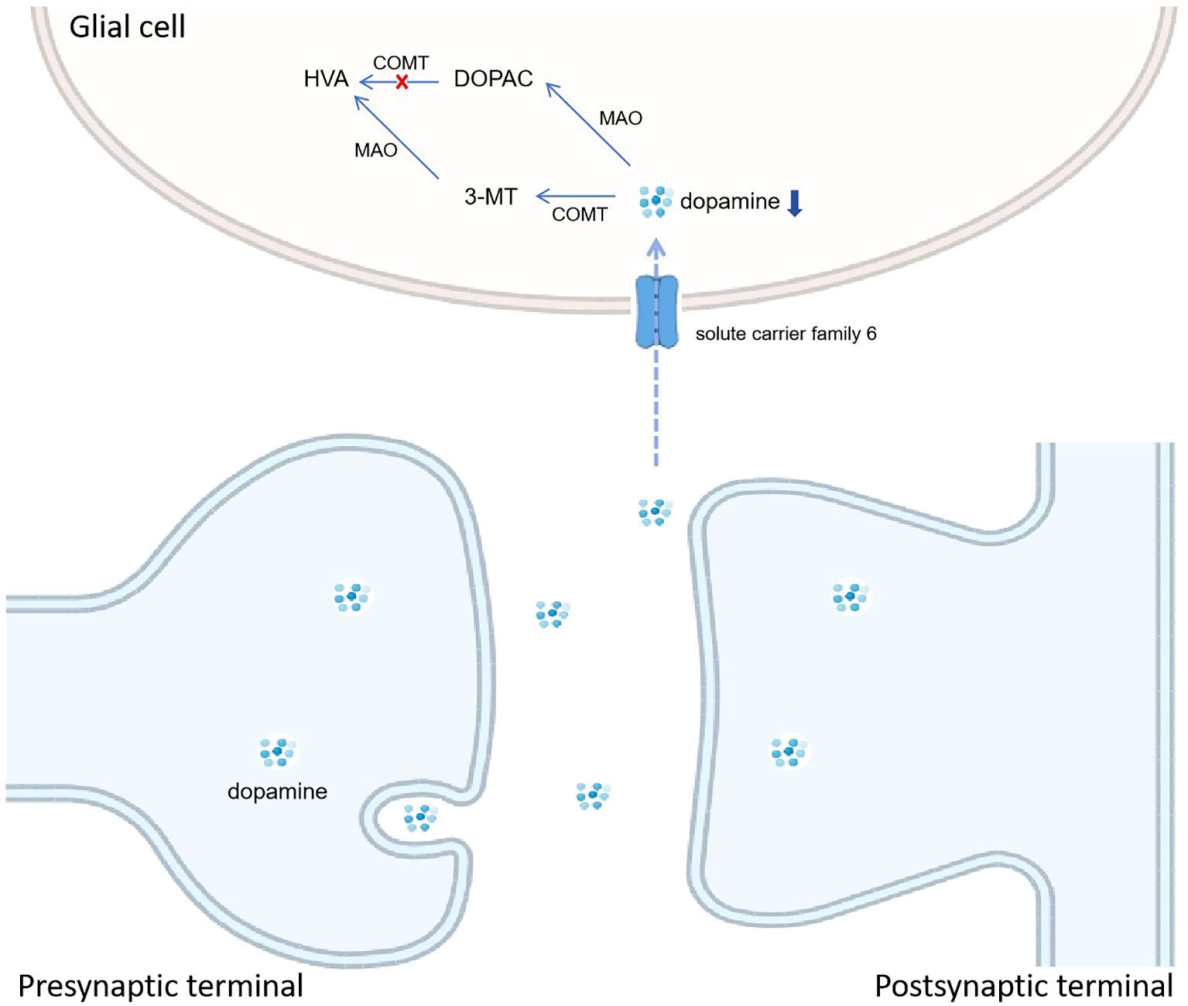
Hypothesized mode pattern of dopamine metabolism in glial cells. We hypothesize the inhibition of catechol-O-methyltransferase (COMT) leads to the accumulation of DOPAC and the downregulation of HAV, which is different from CABM.

Among the differentially expressed neurotransmitters, the upregulated changes in DOPAC levels were of interest to us. As the relevant molecules in dopamine (DA) metabolism, the levels of HAV and 5-MT were downregulated. Meanwhile, DA levels had no statistical difference due to infection status. These neurotransmitters are involved in the tyrosine metabolism pathway. Among them, DA metabolism in glial cells is illustrated in **Figure 4**. DOPAC and 5-MT, both the metabolic products downstream of DA metabolism, are the intermediate products and consequently metabolize to HAV. Our results indicate that the DA-5-MT-HAV pathway was inhibited in PNBM, while the DA-DOPAC pathway was activated. In this process, DOPAC was accumulated but did not then metabolize to HVA. We hypothesized that it would have contributed to the inhibition of catechol-O-methyltransferase (COMT) which can transfer DA to 5-HT and DOPAC to HVA, respectively. Taj and Jamil (2018) also found that in CNS infection by *Listeria monocytogenes*, a high rate of synthesis of DA was observed in comparison with its further degradation into products and the absence of HVA indicated the complete inhibition of COMT. Additionally, although scarce evidence has shown an association between CNS infection and COMT expression, some studies have shown that COMT level might be influenced by inflammation *in vitro* or infection at other organs *in vivo*. Ogburn et al. (2006) found that prostaglandin J2 decreased COMT expression level, inducing the sequestration of COMT into large aggregates and a decline in COMT activity in human neuroblastoma SK-N-SH cells. Additionally, Zhou et al. (2023) demonstrated that colonic COMT levels were significantly decreased and correlated with post-infectious states in diarrhea-predominant irritable bowel syndrome patients as compared to recovered patients and control individuals. Furthermore, some investigations also found associations between *comt* gene variants and infection-promoted risks in certain diseases, such as schizophrenia (Rovira et al., 2022) and HIV infection-related complications (Horn et al., 2017; Saloner et al., 2019). However, we did not test the COMT level or its gene variants in this study. Whether COMT plays an important role in DA metabolism in glial cells needs to be further validated in animal and cytological experiments.

In the bioinformatic analysis, we found that several pathways might be involved in the pathogenesis of PNBM. The results may provide potential information to further investigate how these neurotransmitters exert biological impacts on PNBM. However, after reviewing the published articles on mechanism studies, we found a lack of evidence of a direct association between neurotransmitters and PNBM. We hypothesized that neurotransmitters played a role in biological functions by regulating enzymatic activities. Sokolova et al. performed cytological experiments and found that protein tyrosine kinases, which linked signals from integrins to intracellular signaling pathways, were essential for both bacterial internalization and cytokine secretion by human brain microvascular endothelial cells (Sokolova et al., 2004). Additionally, in an animal model of meningitis, Tumani et al. demonstrated that the inability of hippocampal glutamine synthetase to metabolize excess amounts of glutamate contributed to neuronal apoptosis in the hippocampal formation (Tumani et al., 2000), as these precursors of neurotransmitters could metabolize into different bioactive compounds. After considering the possible differences in mechanism between PNBM and CABM, a further study should be performed on the neurotransmitters with differential expression characteristics in these two diseases from the perspective of mechanism research.

Some limitations should be noticed. First, we performed a preliminary single-center study which contained a limited sample size, and the statistical power value was just 0.053. The results may reflect local information about neurotransmitter changes in PNBM. In future research, the sample size should be augmented using a multi-center, cross-geographical approach to enhance the universality and persuasiveness of the findings. Additionally, because of the low positivity of Gram staining and bacterial culture, we lacked information on pathogenic types. Therefore, the results may be restricted to the general bacterial infection types post-neurosurgery. However, specific types of infections also need to be investigated to find out the particular metabolic style in CNS, which would facilitate a better understanding of the bacterial behavior of certain pathogens and help develop a proper treatment method to prevent infection-related complications. Furthermore, the control samples were extracted from stroke patients without PNBM, and we lacked absolute disease-free samples. Because CSF extraction from healthy subjects may have medical risks, i.e., reducing cranial pressure, causing infection, cerebral hernia, or even death, it is difficult to collect the samples. However, a comparison between the CSF samples from patients with PNBM and healthy subjects would be helpful. Moreover, the result of our study indicated that the potential biological functions of COMT in the pathogenesis of PNBM might be a key point of difference from CABM. To confirm this hypothesis, the pathway of dopamine metabolism in glial cells should be investigated in a mechanism study.

## 5 Conclusion

In summary, we found that the downregulated levels of D-glutamine, Boc-D-Tyr-OH, and L(+)-arginine and upregulated level of DOPAC in CSF had strong diagnostic efficiencies for PNBM in patients with hemorrhagic stroke. The result also offers potential targets in the treatment of PNBM and its complications.

## Data Availability

The original contributions presented in the study are included in the article/**Supplementary Material**. Further inquiries can be directed to the corresponding authors.

## Supplementary material

Supplementary Figure 1 Quality control of targeted metabolomics analysis of neurotransmitters. **(A)** Principle component analysis and **(B)** orthogonal projections to latent structures-discriminant analysis.

Supplementary Figure 2 Expression levels of neurotransmitters in CSF between PNBM cases and infection-free subjects.

Supplementary Table 1 Diagnostic characteristics of neurotransmitters for PNBM.
