# Supplementary figures and images for: Cerebrospinal fluid profiles of targeted metabolomics on neurotransmitters in patients with post-neurosurgical bacterial meningitis
Source: Front Cell Infect Microbiol. 2025 Feb 25;15:1484144. doi: 10.3389/fcimb.2025.1484144 (PMC11893869; doi:10.3389/fcimb.2025.1484144)

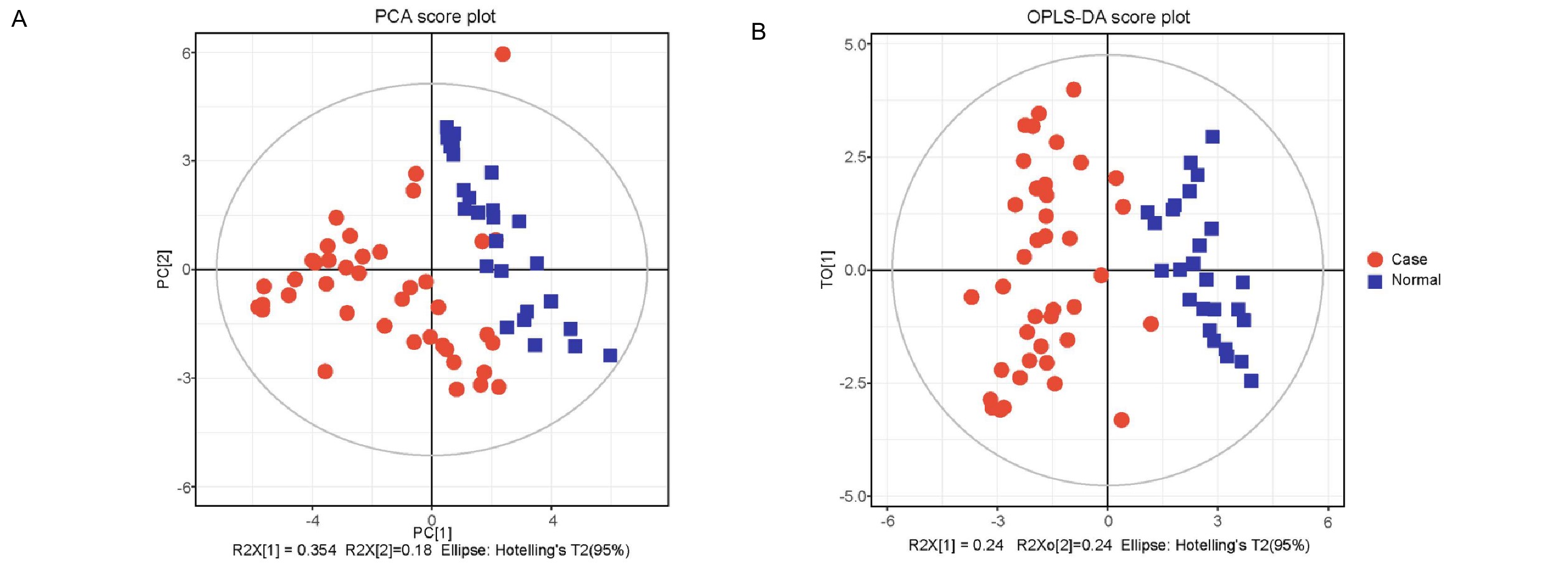

Supplement: Supplementary Figure 1 — Quality control of targeted metabolomics analysis of neurotransmitters. (A) Principle component analysis and (B) orthogonal projections to latent structures-discriminant analysis. [file Image1.jpeg]

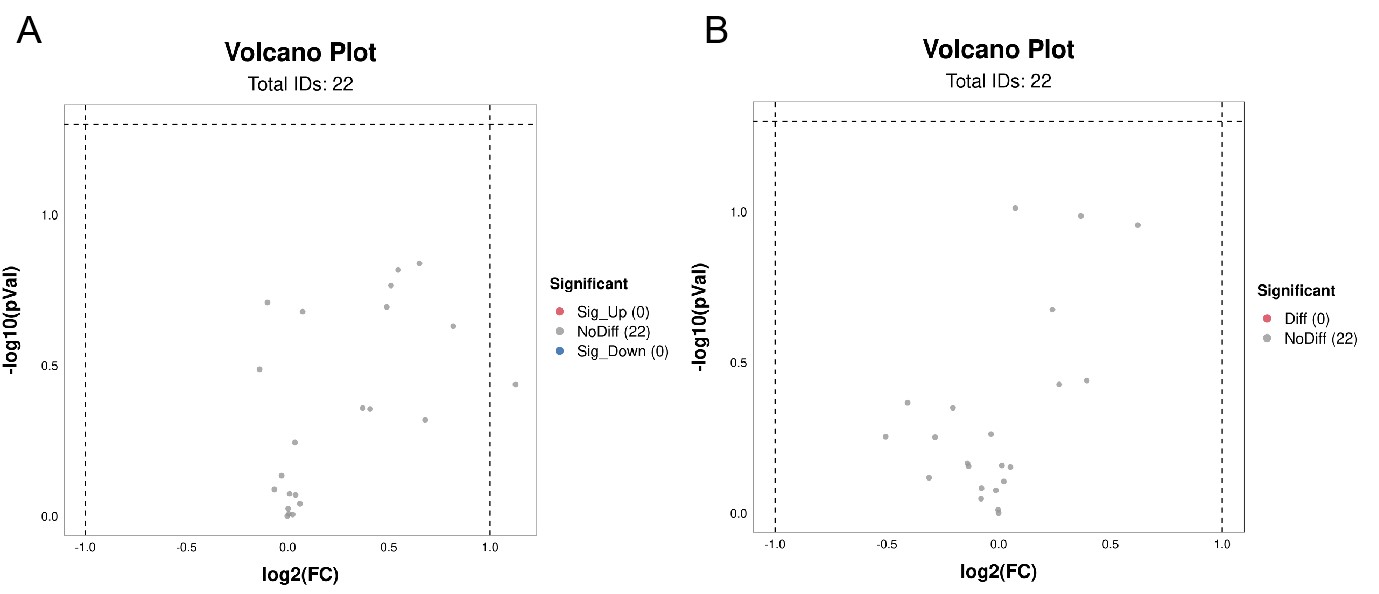

Supplement: Supplementary Figure 2 — Expression levels of neurotransmitters in CSF between PNBM cases and infection-free subjects. [file Image2.jpeg]
