# Supplementary material for: Cerebrospinal fluid profiles of targeted metabolomics on neurotransmitters in patients with post-neurosurgical bacterial meningitis
Source: Front Cell Infect Microbiol. 2025 Feb 25;15:1484144. doi: 10.3389/fcimb.2025.1484144 (PMC11893869; doi:10.3389/fcimb.2025.1484144)
Supplement: Supplementary Table 1 — Diagnostic characteristics of neurotransmitters for PNBM. [file Table1.docx]

**Supplementary Table 1**. Diagnostic characteristics of neurotransmitters for PNBM

|  | AUC | 95% Confidential interval |
| --- | --- | --- |
| D-Glutamine | 1.000 | 1.000-1.000 |
| L-Histidine | 0.721 | 0.591-0.851 |
| Boc-D-Tyr-OH | 0.945 | 0.889-1.000 |
| L(+)-Arginine | 0.942 | 0.883-1.000 |
| D-tryptophan | 0.897 | 0.821-0.974 |
| 5-HIAA | 0.687 | 0.550-0.823 |
| γ-Aminobutyric acid | 0.731 | 0.599-0.863 |
| Serotonin hydrochloride | 0.809 | 0.706-0.911 |
| D-Kynurenine | 0.691 | 0.561-0.820 |
| Kynruenic acid | 0.702 | 0.571-0.833 |
| 3-Methoxytyramine | 0.860 | 0.763-0.957 |
| Acetyl choline | 0.643 | 0.509-0.777 |
| Choline hydroxide | 0.859 | 0.722-0.945 |
| Homovanillic acid | 0.754 | 0.639-0.868 |
| DOPAC | 0.917 | 0.824-1.000 |
